# French national survey on breast cancer care: caregiver and patient views

**DOI:** 10.1007/s12282-024-01576-4

**Published:** 2024-04-18

**Authors:** Christine Rousset-Jablonski, Barbara Lortal, Sophie Lantheaume, Laurent Arnould, Hélène Simon, Anne-Sophie Tuszynski, Mélanie Courtier, Soukayna Debbah, Marc Lefrançois, Sita Balbin, Anne-Sophie Kably, Alain Toledano

**Affiliations:** 1 Centre Léon Bérard, Lyon, France; 2 Unité INSERM U1290 RESHAPE, Lyon, France; 3 Hôpital Femme Mère Enfant, Bron, France; 4 Institut Bergonié, Bordeaux, France; 5 Hôpital Privé Drôme Ardèche, Guilherand-Granges, France; 6 Centre Georges-François Leclerc, Dijon, France; 7 Centre Hospitalier Universitaire, Brest, France; 8 Association Cancer@Work, Anthony, France; 9 Association Jeune Et Rose, Saint-Paul-de-Blaye, France; 10 CMI, Paris, France; 11 Roche SAS, Boulogne-Billancourt, France; 12 Institut de Radiothérapie et de Radiochirurgie Hartmann – ELSAN, Levallois-Perret, France

**Keywords:** Breast neoplasms, France, Health care, Patient views, Cancer survivorship, Good clinical practices

## Abstract

**Purpose:**

To improve the quality of care for patients with breast cancer, an analysis of the health-care pathway, considering feedback from both health-care practitioners (HCPs) and patients, is needed.

**Methods:**

Between 2020 and 2022, we conducted a survey at French breast cancer centers and analyzed information from questionnaires completed by HCPs and patients. We collected information on center organization, diagnostic processes, treatment decisions and modalities, supportive care, patient advocacy groups, and work issues.

**Results:**

Twenty-three breast cancer centers were included and questionnaires completed by 247 HCPs and 249 patients were analyzed. The centers closely followed the legal French framework for cancer treatments, which includes formal diagnostic announcements, multidisciplinary tumor boards, personalized treatment summaries, and supportive care access. HCPs and patients were satisfied with the time to diagnosis (≤ 2 weeks as evaluated by 75% of patients), time to surgery (mean 61 days), time between surgery and chemotherapy (mean 47 days), and time between surgery and radiotherapy (mean 81 days). Fertility preservation counseling for women under 40 years of age was systematically offered by 67% of the HCPs. The majority (67%) of the patients indicated that they had received a personalized treatment summary; the topics discussed included treatments (92%), tumor characteristics (84%), care pathways (79%), supportive care (52%), and breast reconstruction (33%). Among HCPs, 44% stated that reconstructive surgery was offered to all eligible patients and 57% and 45% indicated coordination between centers and primary care physicians for adverse effects management and access to supportive care should be improved, for chemotherapy and radiotherapy, respectively. Regarding patient advocacy groups, 34% of HCPs did not know whether patients had contact and only 23% of patients declared that they had such contact. For one-third of working patients, work issues were not discussed. Twenty-eight percent of patients claimed that they had faced difficulties for supportive care access. Among HCPs, 13% stated that a formal personalized survivorship treatment program was administered to almost all patients and 37% almost never introduced the program to their patients. Compliance to oral treatments was considered very good for 75–100% of patients by 62% of HCPs.

**Conclusions:**

This study provides an updated analysis of breast cancer care pathways in France. Overall, the initial processes of diagnosis, announcement, and treatment were swift and were in agreement with the best care standards. No barriers to accessing care were identified. Based on the study findings, we proposed several strategies to improve the quality of care for patients in supportive care, coordination with primary care physicians, reconstructive surgery, and fertility preservation access.

## Introduction

Worldwide, breast cancer ranks first among cancers in women regarding both incidence and mortality [1].

In France, between 1990 and 2023, the annual number of new breast cancer cases doubled from 30,000 to more than 61,000 cases in 2023. Its incidence is estimated to have increased by 0.3% annually between 2010 and 2023 [2].

Treatments encompass different modalities, from surgery to radiotherapy and systemic treatments, together with supportive care. These treatments, along with long-term survival, necessitate professional interactions within and outside specialized breast cancer teams. Community caregivers including primary care physicians are involved from diagnosis to long-term follow-up of these patients. Patient advocacy groups play an increasingly important role.

The objectives of this survey were to analyze the health-care pathways of French patients with non-metastatic breast cancer, identify good practices and points for improvement, consider feedback from both health-care practitioners (HCPs) and patients, and make proposals to improve quality of care.

## Materials and methods

The survey was conducted at French public and private centers specializing in breast cancer care. A scientific committee was established to organize and control the study and discuss the results. The committee comprised eight members: two patient advocacy group representatives, one oncologist, one pathologist, one gynecologist, one hospital pharmacist, one nurse, and one psychologist. The questionnaires were designed by the scientific committee to capture two types of information: (1) qualitative and semi-quantitative data from HCPs and patients to provide two perspectives of the health-care pathway (questionnaires are provided in the supplementary data) and (2) objective information regarding the duration of steps in the pathway and the type of supportive care provided, based on aggregated data from 10 to 15 consecutive patient charts from each center’s database. The collected information included center organization, diagnostic process, treatment decisions, modalities of surgery, radiotherapy, systemic treatments, and supportive care. Questions regarding coordination between HCPs were asked, and the answers were based on respondent perceptions. Relationships with patient advocacy groups and work issues were also analyzed.

For each breast cancer center involved, the caregiver questionnaire answers were provided by 10 hospital-based HCPs (oncologist, surgeon, nurse, psychologist, radiotherapist, pharmacist, radiologist, pathologist, plastic surgeon, and gynecologist) plus 1 primary care physician. These questionnaires consisted of up to 61 closed-ended questions for some HCPs ((depending on his/her specialty, each HCP was assigned different questions). The answers were compiled using Survey Monkey. The questionnaires were administered by a consultant from CMI to seven HCPs to which the highest number of questions were assigned (oncologists, surgeons, nurses, radiotherapists, pharmacists, psychologists, and primary care physicians) or were self-administered by the other HCPs.

Each center sent a link for the patient questionnaire to a pool of 50 patients representing their center. The patients then filled out the self-administered questionnaire anonymously. These questionnaires consisted of 58 closed-ended questions, whose answers were compiled using Survey Monkey. On average, the patient questionnaires were completed by 11 patients per center.

For each center, objective information was aggregated anonymously at the site level for each item and reported by a reference HCP. For this type of study, formal consent was not required.

The data were collected centrally between December 2020 and January 2022. Information extracted from the questionnaires was tabulated in a descriptive manner.

The centers were the pivotal point of this study. For each center, we established a distribution of answers from the HCPs and patients. This distribution constituted the center’s profile, and the results at the national level were calculated as the average of all center profiles. Thus, in the results presented herein, each center carried the same weight regardless of the number of HCPs and patients who responded per center.

## Results

### Characteristics of the participating centers

Twenty-three centers were involved, all specialized in the management of patients with breast cancer: three university hospitals, five general hospitals, six comprehensive cancer centers, and nine private clinics.

Objective information was received from 22 of the 23 centers; 247 HCPs and 249 female patients provided information through their respective questionnaires.

Fifty-six percent of the centers were organized at a single site for breast cancer treatment. All the centers have tumor boards. The HCPs most commonly involved in tumor boards are surgeons, oncologists, radiotherapists, radiologists, and pathologists. All centers have a pathologist, of whom 65% were specialized in breast cancer. All centers offer supportive care, most often by psychologists, pain management teams, adapted physical activities, beauticians, physiotherapists, dieticians, and social workers. Enhanced recovery after surgery was achieved in 55% of the centers.

Each center monitored an average of 278 patients (minimum 22, maximum 1100, standard deviation [SD] = 312 patients; *N* = 19 centers) during initial treatment (Table 1). However, a significant proportion (42%) of centers treated fewer than 100 patients, whereas 21% managed more than 500 patients. The mean number of followed up patients varied greatly according to the center type: 43 patients in university hospitals, 83 in general hospitals, 623 in comprehensive cancer centers, and 287 in private clinics.

**Table 1:** Patients in treatment per center type

Center type	Average	Min	Max	SD	*N* = number of centers
University hospitals	43	35	50	11	2
General hospitals	83	22	236	87	5
Comprehensive cancer centers	623	500	854	157	4
Private clinics	287	29	1100	352	8

The number of patients followed up after the initial treatment phase was larger (Table 2), with a mean of 521 [min 19, max 2000; SD = 648; *N* = 14 centers] patients per institution.

**Table 2:** Patients in follow-up per center type

Center type	Average	Min	Max	SD	*N* = number of centers
University hospitals	106	50	162	79	2
General hospitals	70	19	121	72	2
Comprehensive cancer centers	1060	506	1674	586	3
Private clinics	537	25	2000	722	7

Forty-seven percent of patients had a breast cancer diagnosis 1 year or less before their participation, 38% between 1 and 5 years, and 15% more than 5 years prior.

### Diagnosis

A breast cancer diagnosis pathway, in which all the necessary steps that each patient would follow for cancer characterization and disease extension, was formalized completely (all steps described) or partially (some steps described) by 72% of the centers.

For breast cancer diagnosis and for announcement of breast cancer diagnosis, the professionals involved, according to the HCPs and patients, are described in Table 3.

**Table 3:** Professionals involved in breast cancer diagnosis and in the announcement of breast cancer according to HCPs and patients

	HCPs (%)	Patients (%)
Breast cancer diagnosis		
Community radiologist	84	65
Primary care physician	78	56
Community gynecologist	72	47
Radiologist of the center	70	44
Announcement of breast cancer diagnosis		
Surgeon	62	23
Primary care physician	48	20
Community gynecologist	48	25
Radiologist	48	29
Oncologist	27	14

HCPs underscored that a specific formal announcement consultation was always made, either at the time of pathological diagnosis (53%) or before the first initial treatment (50%). The HCPs in charge were surgeons (70%), oncologists (48%), nurses (44%), and radiologists (26%).

Complete diagnostic information encompassed patient and clinical characteristics, pathology, tumor biomarkers, and disease extension. It was obtained in less than 4 days for 20% of the HCPs, 4–7 days for 41%, 1–2 weeks for 38%, and more than 2 weeks for 1%. Regarding the time between cancer suspicion and formal diagnosis announcement (histological result), 51% of HCPs felt it was too long for only 0–4% of the patients and 31% for 5–24% of the patients. Most patients (87%) reported that the diagnostic process was rapid; 75% of patients estimated that their delay between mammogram and biopsy was ≤ 2 weeks, 76% of patients were aware that their case was discussed on a tumor board, 8% claimed that it was not, and 16% did not know that a tumor board existed.

The systematically assessed biomarkers were estrogen/progesterone and HER-2 receptors (both had 98% of HCPs), tumor grade (90%), and Ki-67 (89%). The results were available for all cases at the time of the formal announcement consultation.

For 81% of the HCPs, depending on the age of diagnosis, family history, and histopathological characteristics, an oncogenetic consultation was planned when the result could impact treatment decisions.

### Treatments and care

#### Before and including surgery

Neoadjuvant systemic treatment was administered to eligible (as perceived by the responding HCPs) patients as follows: 96–100% patients for 69% HCPs, 75–95% patients for 25% HCPs, and 6% HCPs did not know. The proportion of patients receiving neoadjuvant systemic treatment was 10–20% for 46% HCPs and 20–40% for 25% HCPs. Patients who estimated that they had been treated with neoadjuvant chemotherapy or neoadjuvant hormonotherapy were 33% and 5%, respectively.

Fertility preservation counseling for women under 40 years of age was systematically offered by 67% of the HCPs and upon patient request, by another 9%. Only 5% of the HCPs did not propose this type of counseling, and 19% did not know if there was a fertility preservation pathway integrated into their health-care journey.

**Fig. 1:**
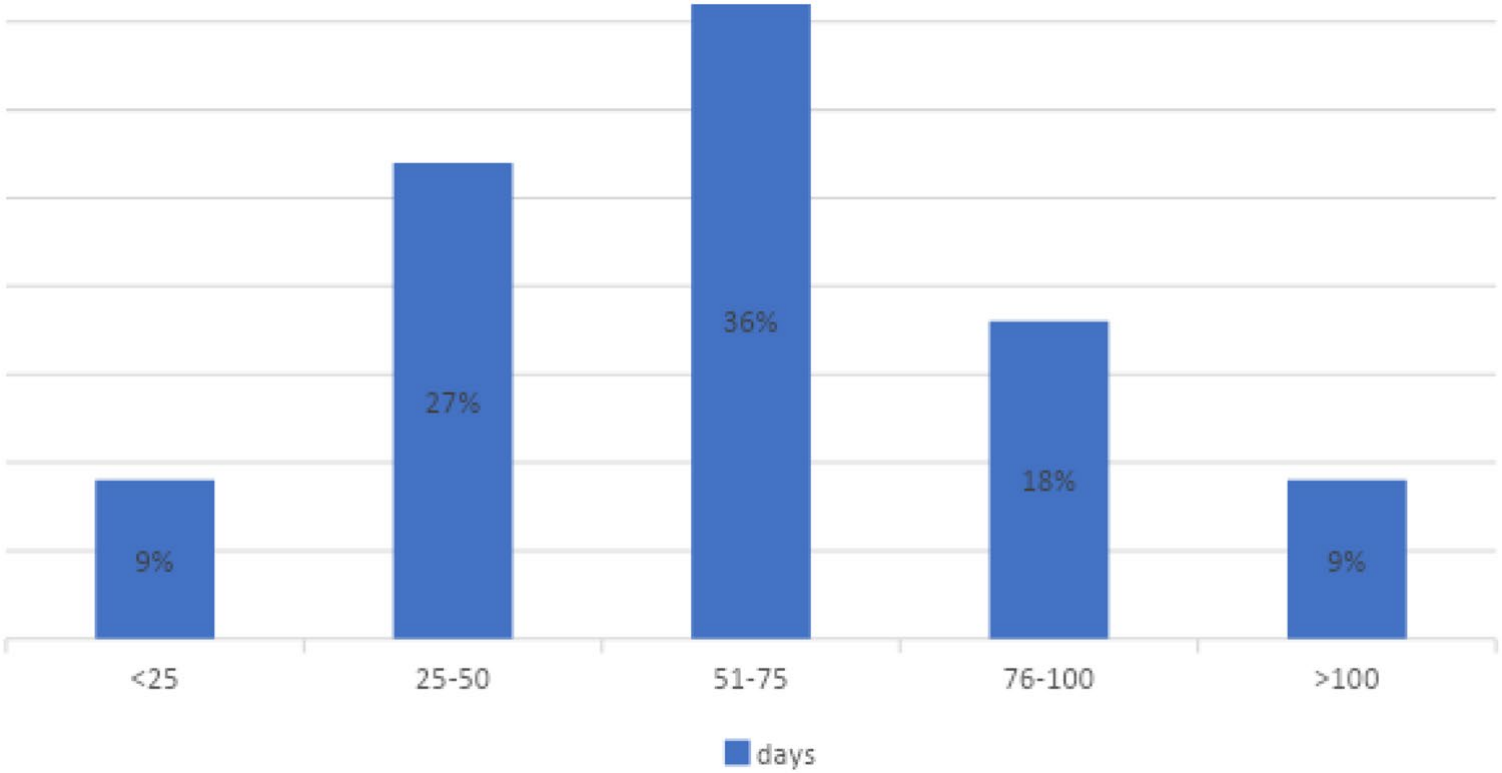
Mean time between first imaging results and surgery

The mean time between the first imaging results and surgery was 61 days (minimum 10, maximum 122 SD = 30 days; *N* = 22 centers). However, we noted large variations in these delays among the participating centers (Fig. 1).

Centers organized at a single site displayed a mean time of 58 days (minimum 18, maximum 122, SD = 31 days; *N* = 15 centers) between imaging and surgery. The centers operating on different sites had a mean delay of 68 days (minimum 10, maximum 99, SD = 29 days; *N* = 7 centers).

Fifty percent of patients benefited from outpatient surgery (63% in comprehensive cancer centers, 53% in private clinics, 45% in general hospitals, and 35% in university hospitals). This was confirmed by 49% of patients who indicated that they underwent day surgery.

Eighty-two percent of patients indicated that surgery was performed in the same institution where they were followed up for breast cancer. Ninety-nine percent of the patients considered that they were well informed about the surgical procedures and 97% considered it swift.

HCPs perceived communication between the surgeons and other HCPs as fairly good: 96% found it good or very good with oncologists, 96% with radiotherapists, and 62% with HCPs in charge of supportive care.

#### After surgery

##### Personalized treatment summary

Seventy-six percent of the HCPs indicated that almost all patient files were discussed on post-surgery tumor boards. In addition, 71% proposed a personalized treatment summary (written document summarizing the various treatments planned) for most patients (75–100%). The HCPs most involved in personalized treatment summaries were oncologists (72%), surgeons (51%), and radiotherapists (52%). The treatment summaries included scheduled treatments (90%), planned visits (50%), supportive care (45%), and imaging (40%).

Sixty-seven percent of patients indicated that they had received a personalized treatment summary: 23% did not receive any personalized treatment summary and 10% did not know. According to the patients, the topics discussed in the personalized treatment summary addressed treatments (92%), tumor characteristics (84%), care pathways (79%), supportive care (52%), and breast reconstruction information (33%).

##### Chemotherapy

Almost all HCPs declared that most adjuvant (or neoadjuvant) chemotherapy sessions were conducted in the center where the patient was followed up. This was confirmed by 92% of the patients. Only 2% of the patients had chemotherapy at home. If offered, 75% of the patients would not have wanted to receive chemotherapy outside the center where they were followed up.

Concerning chemotherapy sessions, 97% of the patients considered that they were well informed and 96% considered the process to be swift. The hormone therapy rates were similar (94% and 95%, respectively).

The mean delay between surgery and the first chemotherapy administration was 47 days (minimum 20, maximum 89, SD = 15 days; *N* = 22 centers).

##### Radiotherapy

Coordination between the oncologist and the radiotherapist was perceived as very good or good by 93% of the HCPs.

Most radiotherapy sessions are performed at the center where the patient is followed up. Seventy-eight percent of HCPs declared that only 0–24% of their patients receive radiotherapy in an institution different from where they were followed up for their breast cancer. This was confirmed by 77% of the patients. Regarding radiotherapy sessions, 100% of the patients considered that they were well informed and 98% considered the process to be swift.

**Fig. 2:**
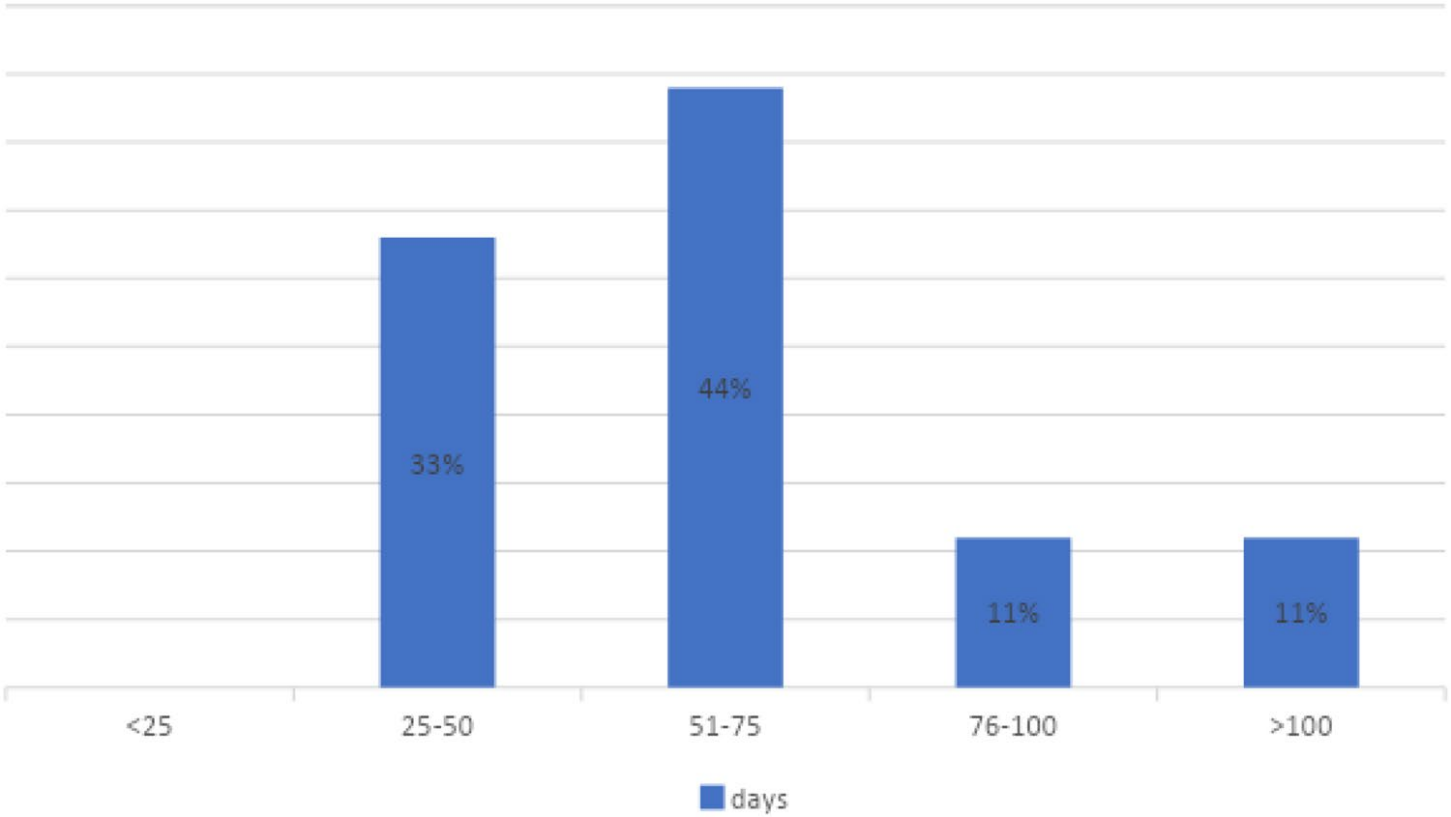
Mean time between surgery and first radiotherapy administration

Seventy percent of the HCPs considered that there were no noticeable delays impacting the radiotherapy sessions. The mean delay between surgery and the first radiotherapy administration was 81 days (minimum 25, maximum 358, SD = 73 days; *N* = 18 centers). However, there were large variations in the timing between surgery and first radiotherapy administration when no adjuvant chemotherapy was administered (Fig. 2).

#### Supportive care and coordination

The centers offered different tools to manage the adverse effects of chemotherapy and radiotherapy: information booklets (72%), direct phone lines (55%), coordinating nurses (54%), patient educational programs (43%), coordination with primary care physicians (34%), and web-based platforms (21%).

Coordination between the centers and the primary care physicians (general practitioner, gynecologist) for adverse effects management and access to supportive care was considered as very good or fairly good for chemotherapy by 24% of HCPs and for radiotherapy by 32%. The HCPs who considered these as “to be improved” were 57% and 45%, respectively.

In addition, the patients evaluated the role of their primary care physicians in the management of their disease. They felt that they were involved in cancer treatment (36%), adverse effects management (15%), and comorbidity management (31%). Nine percent felt that their primary care physicians were not involved: 65% believed that the coordination between the referent HCP of the institution and the primary care physician was good or very good and 35% believed that this relationship needed to be reinforced.

Regarding supportive care, 67% of HCPs indicated that access was excellent for 75–100% of patients, whereas 15% of HCPs considered it excellent for 50–74% of patients and 10% for 5–50% of patients.

The management of supportive care needs and coordination by caregivers as determined by the HCPs and patients is presented in Table 4.

**Table 4:** Management of supportive care needs and supportive care coordination according to HCPs and patients

	Supportive care need		Supportive care coordination	
	HCPs (%)	Patients (%)	HCPs (%)	Patients (%)
Oncologist	74	42	29	26
Nurse responsible for diagnostic announcement	56	10	n.a	n.a
Day-care clinic HCP	52	12	19	12
Patient herself	54	43	19	44
Coordinating nurse	37	9	42	11
Surgeon	36	33	6	16
Radiotherapist	33	5	6	1
Other	27	3	34	0

*n.a.* not applicable

Seventy-two percent of the patients claimed that they did not face difficulties accessing supportive care. However, 28% mentioned the following difficulties: lack of availability of supportive care professional (12%), lack of information (5%), and financial reasons, missing contacts, or other reasons (3% each).

**Fig. 3:**
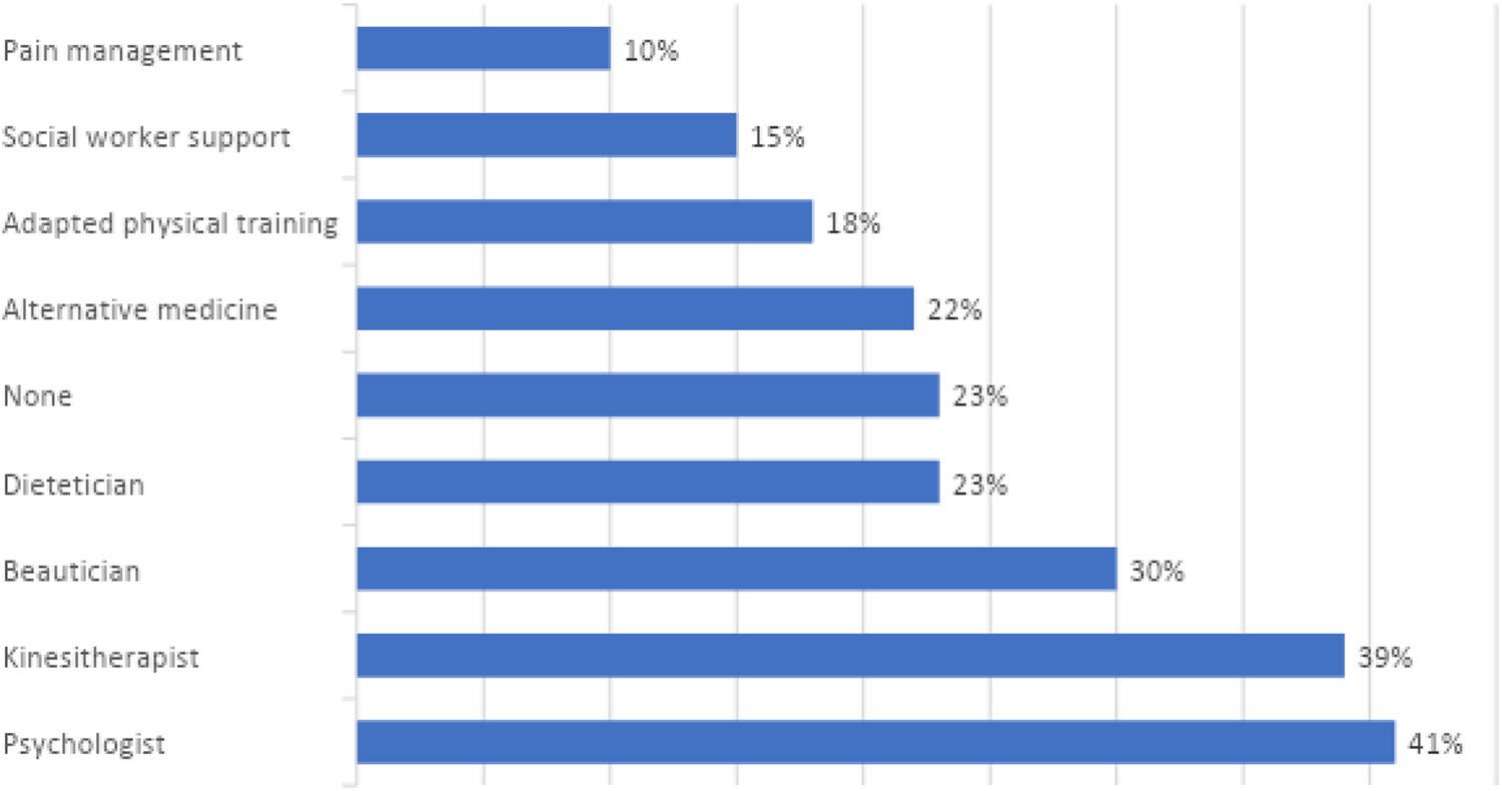
Supportive care options as used by the patients

The various supportive care services used by the patients are presented in Fig. 3.

Ninety-four percent of patients reported that they understood the supportive care proposal and 89% found it swift.

### Patient advocacy groups and work issues

HCPs had contrasting opinions regarding formal contact between their patients and patient advocacy groups: 34% did not know if such contact had occurred, with 32% claiming that there were such contacts for 0–49% of their patients and 34% stating that there were contacts for 50–100% of their patients.

A minority of patients (23%) said that they had formal contact with a patient advocacy group: 15% due to their breast cancer HCP, 7% from their own initiative, and only 1% indicated that contact was through their primary care physicians.

Contact with patient advocacy groups was never made at the time of the diagnostic procedures. It occurred at the time of diagnosis announcement (11%), time of surgery (14%), time of post-surgical treatment (62%), or at the end of treatments (13%).

Patients reported that work issues were discussed with 54% of them and not discussed with 22%, and the question was not applicable for 24%. Among working patients, 30% wanted to maintain professional activities, whereas 70% did not. Patients indicated that the primary person initiating the discussion of work issues was the patient (52%), the oncologist (51%), or the primary care physician (37%). The other HCPs were much less involved. Work issues were discussed at the time of diagnostic procedures (11%), diagnosis announcement (56%), surgery (27%), post-surgical treatment (29%), or at the end of treatments (14%).

Sixty-eight percent of patients considered that they had been coached to maintain their professional activities and was due to the attention of their employer (31%), they could benefit from part-time therapy (24%), they could adapt their time at work (9%), or they were coached by a social worker (4%).

Breast cancer had no impact on earnings for 44% of the working patients and 25% indicated an earning reduction of up to 25% and 31% of 26% or more.

### Personalized survivorship treatment program

The personalized survivorship treatment program takes over from the personalized care program at the end of the initial treatment. This allows the patient to integrate her follow-ups into her daily life, adapted to her needs.

For reconstructive surgery, 44% of the HCPs estimated that it was offered to almost all eligible patients. However, 34% believed that it was offered to 50–95% of eligible patients. Thirty-six percent of patients said that they benefited from reconstructive surgery.

According to 13% of the HCPs, a formal personalized survivorship treatment program was administered to almost all patients: 37% of them almost never proposed the program to their patients and 36% did not know. Few patients (20%) believed that they had received a formal personalized survivorship treatment program: 37% believed they did not and 43% did not know what it is.

The HCPs deemed long-term follow-up after the first 5 years was ensured by the community gynecologist (76%), primary care physicians (66%), oncologists (58%), surgeons (51%), and radiotherapists (49%). For 75% of the HCPs, follow-ups also involved physicians who were external to the center.

Sixty-two percent of HCPs considered compliance with oral treatments to be very good for 75–100% of their patients. The patients judged their compliance with long-term oral treatments as excellent (62%), good (8%), poor (7%), or very bad (23%).

Of the HCPs, 56% claimed that their patients were not lost to follow-up, whereas 24% believed that 5–25% of their patients were lost to follow-up; of these, 79% believed that being lost to follow-up could negatively impact prognosis.

Twenty-seven percent of patients indicated that they were not provided any means of follow-up by their breast cancer center.

## Discussion

Our study found that breast cancer is diagnosed mainly at the community level by primary care physicians or community specialists such as gynecologists or radiologists. A breast cancer diagnosis is announced by a large panel of caregivers, both in the community and in the hospital. The observed timings at the diagnostic steps (61% of HCPs giving a maximum of 1 week for full diagnostic information) appear reasonably short [3]. Even slightly longer delays (up to 2 weeks for 38% of HCPs) should not impact the diagnostic quality, treatments, and follow-up. Better patient information regarding necessary delays in achieving a full diagnostic process would reduce patient anxiety and expectations.

The centers closely follow the procedures of formal announcements, multidisciplinary tumor boards [4], personalized treatment summaries, and access to supportive care. This agrees with the legal framework set for cancer treatment in 2007 [5, 6]. The centers’ organization is also in agreement with the requirements of a specialist breast center as defined by the EUSOMA in 2020 [7]. These guidelines underline the need for multidisciplinary and patient-centered pathways from diagnosis to treatment and survival. The French breast cancer centers participating in our study were in good agreement with the EUSOMA recommendations for most topics, such as the minimum number of new patients per year, patient care pathway, tumor board organization, communication of diagnostic and treatment plans with patients, and patient advocacy. However, the minimum caseload of core tumor board members was not addressed in our study.

All patients had a formal announcement consultation, which is a key component for disease acceptance and full patient cooperation [8]. We recommend giving enough time to discuss social aspects and potential working problems during this consultation. Eighty-five percent of patients knew what a tumor board was, indicating that most patients were well informed.

Patients expressed very high degrees (> 95%) of satisfaction with the information and timing of surgery, chemotherapy, and radiotherapy. This high level of patient satisfaction is consistent with what has recently been measured in other developed countries [9, 10].

We found reasonably short delays between the first imaging results and surgery (mean 61 days), between surgery and chemotherapy (mean 47 days), and between surgery and radiotherapy (mean 81 days). In large non-randomized series, increased time to surgery is related to lower overall and disease-specific survival [11]. The centers organized at a single site displayed a mean time of 58 days between imaging and surgery. This was shorter than the 68 days observed in centers operating at different sites. Approximately 22% of the centers had a delay in radiotherapy exceeding 76 days. National recommendations state that the delay should be less than 12 weeks (84 days) [12]; thus, centers that regularly exceed this time should review this with their organization.

As only 67% of HCPs proposed fertility preservation counseling for women under 40 years of age, there is room for improvement [13–15]. An alert for women with childbearing potential could be implemented, and discussions about fertility, contraception, and sexual health concerns should be engaged, preferably before surgery [16–19].

Day surgery was proposed for half of the patients. Day surgery is known to lower hospital costs and to foster high patient satisfaction [20–22]. One of the main hurdles is the distance between a patient’s home and hospital. The proportion of day surgeries might increase using distant follow-up tools as well as a greater use of hospital hotels. However, a short hospital stay should not deny patients full access to supportive care.

After surgery, 71% of the centers proposed a personalized treatment summary for most, which can further improve patient knowledge about their diagnosis and treatments [23, 24]. A personalized treatment summary should be regularly and widely shared during the post-surgery period and follow-up, as well as with external caregivers.

When adjuvant chemotherapy was indicated, patients preferred to be treated in breast cancer centers. The timing of chemotherapy was considered satisfactory by both patients and HCPs. To increase the proportion of patients benefiting from chemotherapy-day hospitals, organizational improvements should focus on early chemotherapy prescriptions, administrative pre-admission, and calling patients to ensure the availability of biological results. Remote consultations (telemedicine) with patients may also facilitate this process [25, 26].

Traditionally, oncology specialists deliver follow-up care at breast cancer centers. The number of oncologists in France is still rising [27]. However, with the increase in hospital costs and improved treatments resulting in a larger number of survivors, a specialist-only model may not be optimal. The primary care physician should be increasingly involved in patient follow-up [28–30]. The difficulty in involving primary care physicians may be explained by patients’ preference for hospital follow-up and concerns among patients and hospital specialists regarding the oncology knowledge of primary care physicians [31, 32].

When it comes to the management of adverse effects and access to supportive care, approximately 50% of HCPs determined room for improvement regarding coordination with primary care physicians. Thirty-six percent of the patients believed that this relationship needed to be reinforced. Relationships can be strengthened through high-level initiatives such as breast cancer courses and direct phone lines. Telemedicine may also help manage the adverse treatment effects [33].

Supportive care was readily available to both patients and HCPs. Some had already taken communication initiatives with patients for supportive care, including flyers, goods, and social network pages.

Contact between patients with breast cancer and patient advocacy groups is far from systematic. One-third of the HCPs claimed that they had no idea if such contact had occurred, and less than one-quarter of the patients had formal contact with a patient advocacy group. We believe that support from these groups is important, particularly after the primary treatment phase because it facilitates access to supportive care and correct supportive care use. This support also helps patients to maintain social relationships when loneliness can occur. Proactive presentation of patient advocacy groups by HCPs should also increase.

Work issues appear to have been discussed insufficiently. Having regular discussions with patients is a good practice. Some of us have developed workshops to counsel patients about work-related issues. The primary care physician may assist the patient in returning to work [34]. Attention should be paid to the adverse effects of long-term hormone therapy, which can cause patients to drop out of professional activities. Half of the patients reported a drop in their work revenue. Interestingly, our survey showed that only 30% of working patients wanted to return to work after breast cancer diagnosis.

Only 44% of the HCPs believed that reconstructive surgery should be proposed for all eligible patients. This is in line with a large French observational study that showed a high proportion (66%) of women did not undergo breast reconstruction after mastectomy [35]. Although access to reconstruction was considered easy by the majority of patients, it appears that more HCPs should present this option to patients. Some centers have formalized patient documentation on reconstructive surgery and have specific consultations after radiotherapy.

Compliance with long-term adjuvant hormone therapy is important. Even if 70% of patients say they are compliant, studies have demonstrated an overestimation of patient compliance and the impact of poor compliance on oncological endpoints [36]. Therefore, improving patient compliance with oral drug treatments is essential. The quality of relationships with primary care physicians may contribute to better compliance [37]. Thus, nurses and community pharmacists can foster patient compliance.

Among its strengths, the study was designed by a multidisciplinary body of HCPs. A large number of centers were involved, which were representative of the different types of hospital organizations. The results were validated using a double approach with 247 HCPs and 249 patients. For most topics, information from HCPs and patients could be superimposed.

This survey has several limitations. The participating centers were not randomly selected and thus might have had a higher standard of care than other centers. HCPs reports on their center organization were declarative and not confirmed by objective data from other sources. Patient satisfaction was assessed through feedback on received information and delays. Formal patient-reported outcome tools were not used. A description of the psychometric properties of the questionnaire designed by the scientific committee to check the quality of completion, validity, and reliability of the measurement tool was not included in the framework of this survey.

## Conclusions

Our study was based on information obtained from patients with breast cancer and specialized HCPs. We identified improvements and proposed solutions for domains where improvement can be made such as supportive care, coordination with primary care physicians, reconstructive surgery, and access to fertility preservation.

## Data Availability

The authors confirm that the data supporting the findings of this study are available on demand at contact@cmistrategies.com.
